# Viricidal Efficacy of a 405‐nm Environmental Decontamination System for Inactivation of Bacteriophage Phi6: Surrogate for SARS‐CoV‐2

**DOI:** 10.1111/php.13798

**Published:** 2023-03-16

**Authors:** Lucy G. Sinclair, Zornitsa Ilieva, Georgina Morris, John G. Anderson, Scott J. MacGregor, Michelle Maclean

**Affiliations:** ^1^ Department of Electronic & Electrical Engineering, The Robertson Trust Laboratory for Electronic Sterilisation Technologies (ROLEST) University of Strathclyde Glasgow UK; ^2^ Department of Biomedical Engineering University of Strathclyde Glasgow UK

## Abstract

The highly transmittable nature of SARS‐CoV‐2 has increased the necessity for novel strategies to safely decontaminate public areas. This study investigates the efficacy of a low irradiance 405‐nm light environmental decontamination system for the inactivation of bacteriophage phi6 as a surrogate for SARS‐CoV‐2. Bacteriophage phi6 was exposed to increasing doses of low irradiance (~0.5 mW cm^−2^) 405‐nm light while suspended in SM buffer and artificial human saliva at low (~10^3–4^ PFU mL^−1^) and high (~10^7–8^ PFU mL^−1^) seeding densities, to determine system efficacy for SARS‐CoV‐2 inactivation and establish the influence of biologically relevant suspension media on viral susceptibility. Complete/near‐complete (≥99.4%) inactivation was demonstrated in all cases, with significantly enhanced reductions observed in biologically relevant media (*P* < 0.05). Doses of 43.2 and 172.8 J cm^−2^ were required to achieve ~3 log_10_ reductions at low density, and 97.2 and 259.2 J cm^−2^ achieved ~6 log_10_ reductions at high density, in saliva and SM buffer, respectively: 2.6–4 times less dose was required when suspended in saliva compared to SM buffer. Comparative exposure to higher irradiance (~50 mW cm^−2^) 405‐nm light indicated that, on a per unit dose basis, 0.5 mW cm^−2^ treatments were capable of achieving up to 5.8 greater log_10_ reductions with up to 28‐fold greater germicidal efficiency than that of 50 mW cm^−2^ treatments. These findings establish the efficacy of low irradiance 405‐nm light systems for inactivation of a SARS‐CoV‐2 surrogate and demonstrate the significant enhancement in susceptibility when suspended in saliva, which is a major vector in COVID‐19 transmission.

## Graphical abstract

The COVID‐19 pandemic has increased the necessity for novel strategies to safely decontaminate public areas. This study investigates the efficacy of a low irradiance 405‐nm light environmental decontamination system for the inactivation of bacteriophage phi6, as a surrogate for SARS‐CoV‐2. In addition, bacteriophage phi6 was exposed to low irradiance (~0.5 mW cm^−2^) 405‐nm light while suspended in SM buffer and artificial human saliva, to establish the influence of biologically relevant suspension media on viral susceptibility. Results indicated complete/near‐complete (≥99.4%) inactivation in all cases, with significantly enhanced reductions observed when suspended in saliva (*P* < 0.05), which is a major vector in COVID‐19 transmission.

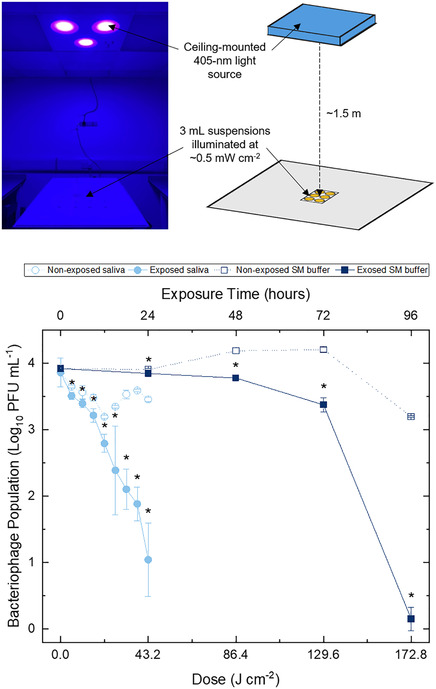

## INTRODUCTION

Severe acute respiratory syndrome coronavirus 2 (SARS‐CoV‐2) is a novel single‐stranded enveloped RNA coronavirus which instigated the ongoing coronavirus disease 2019 (COVID‐19) pandemic and has caused, at the time of writing, over 578 million infections and 6.4 million deaths worldwide (1); the highest number of global deaths in comparison with all other pandemics in the last century (2).

The disease is highly contagious (3) and person‐to‐person transmission is believed to occur predominantly through contact with oral–nasal respiratory secretions and airborne droplets generated from infected individuals (4, 5). Consequently, it is well‐recognized that poorly ventilated indoor communal spaces provide a significant risk of SARS‐CoV‐2 transmission (6) and multiple COVID‐19 outbreaks have been reported within crowded closed settings in which people are in close proximity for extended periods of time (7). In addition, the virus has been shown to survive and remain viable in the environment for multiple days, and in some cases weeks, on various surfaces and fomites (8, 9, 10), with the risk of exposure substantially increased at higher viral loads (11).

An enhanced understanding of the role of closed communal environments as a source of SARS‐CoV‐2 transmission has focused attention on the importance of environmental cleaning and disinfection as a means of reducing disease spread. Established techniques for whole‐room decontamination of public environments such as ultraviolet radiation has demonstrated sufficient efficacy toward the disinfection of SARS‐CoV‐2 (12, 13, 14, 15); however, they are broadly limited to infrequent use in unoccupied and sealed environments due to their harmful radiation effects and long‐term material degradation upon repeated exposure (16, 17). Consequently, novel methods of environmental decontamination to augment current SARS‐CoV‐2 infection control procedures are continuously being sought.

A recent development in whole‐room environmental decontamination is the use of visible violet‐blue light in the region of 405‐nm which has been shown to provide safe and continuous decontamination of occupied whole‐room environments (18, 19). These ceiling‐mounted environmental decontamination systems (EDS) emit white light blended with increased levels of antimicrobial 405‐nm light, which inactivates microorganisms through ROS‐mediated oxidative damage initiated by the photoexcitation of endogenous porphyrin molecules (20, 21). The bactericidal efficacy of the 405‐nm light EDS is well established, with evidence demonstrating its ability to successfully reduce bacterial contamination levels within patient isolation rooms by up to 86% when used in conjunction with routine cleaning procedures (18, 22, 23, 24) and, more recently, significantly reduce rates of surgical site infections when used in operating theaters (25).

By comparison, the viricidal properties of the 405‐nm EDS are less understood. Unlike bacterial cells, viruses do not contain porphyrins within their morphological structures, so viral 405‐nm light inactivation is instead thought to be due to an alternative mechanism. Tomb *et al*. previously demonstrated that inactivation of non‐enveloped viruses is possible in the absence of photosensitizers, but at much higher doses than that required by bacteria (~10 times greater doses required for a 1 log_10_ reduction (26)), suggesting the inactivation effect observed is possibly due to exposure to the low‐level UV‐A photon output (380–390‐nm) at the tail‐end of the 405‐nm LED emission spectrum (27, 28). The authors additionally noted that viral susceptibility can be increased when suspended in photosensitive media including artificial saliva (27, 28); which was further corroborated by Kingsley *et al*. (29), who demonstrated greater inactivation of non‐enveloped Tulane virus when exposed in the presence of singlet oxygen enhancers.

In light of the recent COVID‐19 pandemic, establishing the efficacy of 405‐nm light for the inactivation of SARS‐CoV‐2 is of significant research interest. Rathnasinghe *et al*. (30) recently demonstrated successful reductions in SARS‐COV‐2 upon exposure to low irradiance (0.035–0.6 mW cm^−2^) 405‐nm light, and additionally highlighted the increased susceptibility of lipid‐enveloped viruses in comparison with non‐enveloped viruses (identical irradiations achieved 2.3 log_10_ reductions in SARS‐COV‐2 after 8 h and just 0.1 log_10_ reductions in a non‐enveloped RNA virus after 24 h); suggesting the lipid envelope itself may instigate ROS production. Other studies have similarly demonstrated the susceptibility of SARS‐CoV‐2, or an appropriate surrogate, to 405‐nm light inactivation presented on surfaces and in liquid media, both in the presence and absence of photosensitizers (31, 32, 33, 34, 35). Although highly encouraging, these studies have primarily demonstrated inactivation using 405‐nm light at high irradiances (≥78.6 mW cm^−2^) or at low irradiances delivered at a very short distance (~2.3–25.4 cm) from the sample surface (30, 31, 32, 33, 34, 35), however, it is of great importance to determine whether inactivation of SARS‐CoV‐2 can be achieved under conditions which more accurately represent those which would be safely and practically implemented for environmental decontamination of communal areas.

The aims of this study were therefore to investigate the antiviral efficacy of 405‐nm light under conditions representative of those implemented for whole‐room decontamination of occupied environments. A ceiling mounted 405‐nm light prototype EDS (as used in clinical environmental decontamination studies (18, 19, 22, 23, 24)) was used to illuminate samples of bacteriophage phi6—a surrogate for SARS‐CoV‐2—using low irradiance 405‐nm light (0.5 mW cm^−2^) at a distance of 1.5 m. Inactivation kinetics were established with the phage suspended in minimal media, to evaluate the effect of direct interaction between the light and the phage and also with the phage suspended in artificial saliva in order to better replicate how the phage would interact with the light treatment when within respiratory secretions and droplets, as would be the case with clinical transmission. Comparison of the antiviral efficacy and germicidal efficiency of higher irradiance (50 mW cm^−2^) 405‐nm light sources for the inactivation of bacteriophage phi6 is also provided. The results provide a means of evaluating the potential of this environmental decontamination technology to be used as a method of controlling transmission of SARS‐CoV‐2 within occupied healthcare settings and other public areas.

## MATERIALS AND METHODS

### Preparation of bacteriophage and host bacterium

Bacteriophage phi6 (DSM 21518) and its host bacterium *Pseudomonas syringae* (DSM 21482) (Leibniz‐Institute DSMZ German Collection of Microorganisms and Cell Cultures GmbH, Germany) were used in the study. For experimental use, *P. syringae* was inoculated in 100 mL Tryptone Soya Broth (TSB; Oxoid Ltd.) and cultured at 25°C under rotary conditions (120 rpm) for 18–24 h to obtain a bacterial cell density of approximately 1 × 10^9^ colony‐forming units (CFU) mL^−1^.

Bacteriophage phi6 was purchased as a 1 mL liquid suspension of bacteria‐free lysates in the host's growth medium. This liquid suspension was propagated and a high titre phage stock solution (3 × 10^10^ plaque‐forming units (PFU) mL^−1^) was prepared using a plate lysis and elution method. Briefly, the phage suspension was serially diluted in SM buffer (50 mm Tris HCl, 100 mm NaCl, 8 mm MgSO_4_ and 0.01% gelatin; G‐Biosciences) and 100 μL of each dilution was mixed with 100 μL of an overnight *P. syringae* TSB culture, which were then co‐incubated at 25°C for 10 min to facilitate phage attachment to cells. Post‐incubation, suspensions were mixed with 3 mL sterile soft enriched agar (TSB with 0.6% Agar Bacteriological Oxoid Ltd.), 5 mm MgSO_4_ (Thermo Scientific) and 5 mm CaCl_2_ (Thermo Scientific) and then poured onto the centre of an enriched TSB agar plate (TSB with 1.5% Agar Bacteriological, 5 mm MgSO_4_ and 5 mm CaCl_2_), swirled and incubated at 25°C for 18–24 h. Post‐incubation, 5 mL SM buffer was added to the plate with confluent lysis, which was then stored on a platform shaker for 40 min with slow agitation. The lysate was then filtered through a cellulose acetate membrane with a 0.2 μm pore size to remove bacterial debris, and the phage stock stored at 4°C for experimental use.

### Exposure of bacteriophage to 405‐nm light

**Figure 1:**
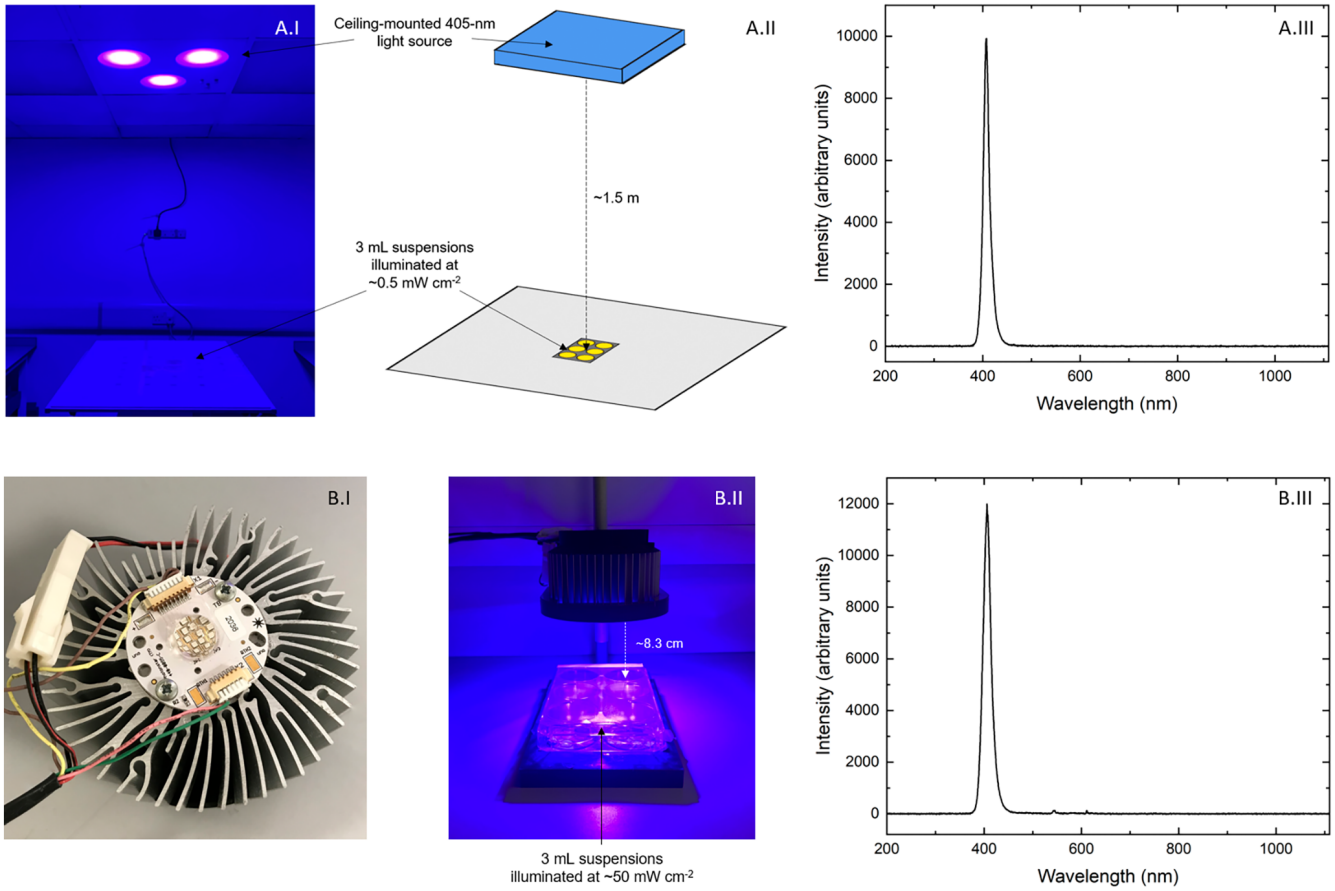
Light sources for exposure of bacteriophage phi6: (A.I) the 405‐nm EDS, (A.II) diagrammatic representation of the experimental arrangement, with the EDS being used in “405‐nm‐only” mode, and (A.III) emission spectra of the 405‐nm output of the EDS; and (B.I) a single 405‐nm LED array, (B.II) experimental arrangement and (B.III) emission spectra of the 405‐nm output of the LED array. All emission spectra data were captured using an HR4000 spectrometer (Ocean Optics, Germany) and Spectra Suite software version 2.0.151. [Colour figure can be viewed at wileyonlinelibrary.com]

The light source used for low irradiance bacteriophage exposures was a prototype ceiling‐mounted 405‐nm light EDS (Fig. 1A.I,A.II), developed at the University of Strathclyde, UK (36). As described previously (18, 19, 22, 23, 24), the light system produces antimicrobial light, generated from a matrix of light‐emitting diodes (LEDs) which emit violet‐blue light with a narrow spectral profile centered on 405‐nm with full‐width half‐maximum (FWHM) of 14‐nm (Fig. 1A.III). For practical deployment, white LEDs are also incorporated into the system so that the illumination effect is predominantly white, however for this study the white LEDs remained off.

For higher irradiance bacteriophage exposures, a single 405‐nm LED array was used (ENFIS PhotonStar Innovate UNO 24 LED array [PhotonStar Technologies Ltd]; Fig. 1B.I). The array was mounted on a polyvinyl chloride housing (Fig. 1B.II), and emitted 405‐nm light (16‐nm FWHM; Fig. 1B.III).

Bacteriophage phi6 was suspended at low (~10^3–4^ PFU mL^−1^) and high (~10^7–8^ PFU mL^−1^) seeding densities in SM buffer and artificial human saliva (1 L sterile H_2_O, 5.29 g/L NaHCO_3_, 0.88 g/L NaCl, 1.36 g/L K_2_HPO_4_, 0.48 g/L KCl, 2000 IU α‐amylase and 2 g/L mucin from porcine stomach (28)). For exposure, 3 mL suspensions (*n* > 4) were positioned either 1.5 m below the 405‐nm light EDS, giving an incident irradiance of approximately 0.5 mW cm^−2^ at the sample surface, or 8.3 cm below the single array system, giving an incident irradiance of approximately 50 mW cm^−2^ at the sample surface. No significant increase in sample temperature was recorded with either high or low irradiance exposures. All irradiance values were measured using a radiant power meter and photodiode detector (LOT Oriel). Samples were exposed to increasing doses of 405‐nm light, with control samples held under standard laboratory lighting for equivalent exposure durations (“ambient light controls”). To comparatively assess the impact of ambient light exposure on bacteriophage survival, additional control samples were held in complete darkness for the maximum duration of 405‐nm light exposures (“dark controls”). Post‐exposure, surviving phage populations were determined *via* co‐incubation with *P. syringae* through a double‐agar overlay method: 100 μL of exposed and control samples, diluted in SM buffer if required, were mixed with 100 μL of an overnight *P. syringae* TSB culture and 3 mL sterile soft enriched agar and then poured onto a 90‐mm enriched TSB agar plate. The plates were swirled, left to set and then incubated at 25°C for 18–24 h before enumeration of the plaques (PFU mL^−1^).

## RESULTS

**Figure 2:**
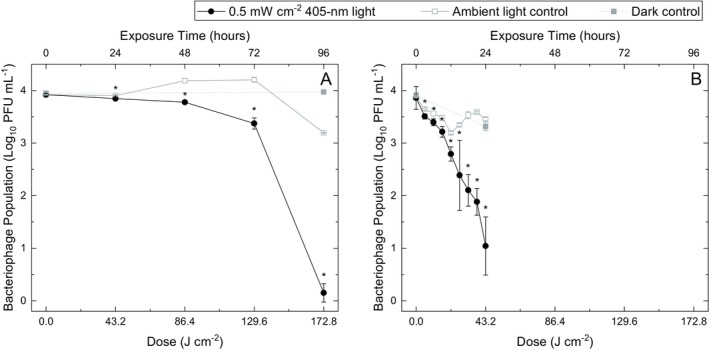
Inactivation of bacteriophage phi6 suspended in (A) SM buffer and (B) artificial human saliva at population densities of 10^3–4^ PFU mL^−1^ upon exposure to increasing doses of 405‐nm light at an irradiance of approximately 0.5 mW cm^−2^. Each data point represents the mean value ± SD (*n* ≥ 4). Asterisks (*) indicate significant differences between exposed and non‐exposed phi6 populations (two sample *t*‐test; *P* ≤ 0.05, Minitab Statistical Software v19). [Colour figure can be viewed at wileyonlinelibrary.com]

**Figure 3:**
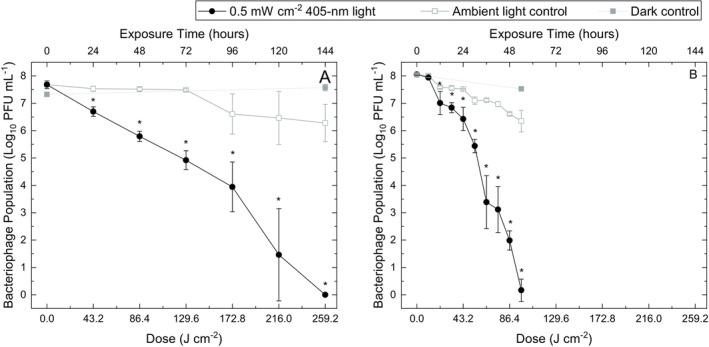
Inactivation of bacteriophage phi6 suspended in (A) SM buffer and (B) artificial human saliva at population densities of 10^7–8^ PFU mL^−1^ upon exposure to increasing doses of 405‐nm light at an irradiance of approximately 0.5 mW cm^−2^. Each data point represents the mean value ± SD (*n* ≥ 4). Asterisks (*) indicate significant differences between exposed and non‐exposed phi6 populations (two sample *t*‐test; *P* ≤ 0.05, Minitab Statistical Software v19). [Colour figure can be viewed at wileyonlinelibrary.com]

Results for the inactivation of bacteriophage phi6 using 0.5 mW cm^−2^ 405‐nm light demonstrate significant inactivation (*P* < 0.05) of low‐ and high‐density populations when exposed in both SM buffer and artificial saliva (Figs. 2 and 3, respectively).

At both low and high seeding densities, susceptibility was shown to be significantly enhanced when bacteriophage populations were exposed while suspended in artificial saliva compared to SM buffer (*P* ≤ 0.05). At low density, exposure to 43.2 J cm^−2^ resulted in a maximum 2.41 log_10_ reduction in artificial saliva, compared with just 0.06 log_10_ reduction in SM buffer. For exposures in SM buffer, a greater dose of 172.8 J cm^−2^ was required to achieve a maximum 3.05 log_10_ reduction. Similarly, at high density, a dose of 97.2 J cm^−2^ achieved a maximum 6.18 log_10_ reduction in artificial saliva, compared with a greater dose of 259.2 J cm^−2^ required to achieve a maximum 6.28 log_10_ reduction in SM buffer. Collectively, results demonstrate that 405‐nm light inactivation was 2.6–4 times more effective when the bacteriophage was suspended in saliva compared with SM buffer.

As hypothesized, the doses/treatment durations required to achieve complete/near‐complete inactivation (approximately ≤1 log_10_ populations remaining) of phi6 populations were greater when exposed at high density as opposed to low density, however, results indicate that the dose required to achieve an approximate 1 log_10_ reduction in artificial saliva was the same when exposed at both low‐ and high‐seeding densities (21.6 J cm^−2^; 12 h). This differed when exposed in minimal SM buffer media, with an approximate 1 log_10_ reduction being achieved with 43.2 J cm^−2^ (24 h) in high‐density populations but requiring >129.6 J cm^−2^ (>72 h) in low‐density populations.

With regards to control populations held in ambient lighting, natural decay was evident over the extended exposure times for all phage suspensions (*P* ≤ 0.05), with decay of the populations in saliva more apparent: at low density, a 0.38 log_10_ reduction was observed following 96 h in SM buffer, compared to a 0.61 log_10_ reduction after just 24 h in saliva. Similarly, at high density, a 1.10 log_10_ reduction was observed after 144 h in SM buffer, compared to a 1.70 log_10_ reduction after 54 h in saliva. In contrast, control populations held in complete darkness demonstrated no significant decay over the extended durations in SM buffer (*P* > 0.05) and although decay was demonstrated in saliva (*P* ≤ 0.05), reductions were still significantly lower than that observed for samples held in ambient lighting. Following 24 h for low‐density populations and 54 h for high‐density populations, 0.61 and 1.70 log_10_ reductions were demonstrated in ambient lighting, respectively, in comparison with 0.60 and 0.51 log_10_ reductions demonstrated in complete darkness, respectively (*P* ≤ 0.05). These findings suggest that, as expected, standard room lighting was also able to induce some photoexcitation—albeit at a significantly lower rate than that of 405‐nm light (27).

A comparison of the inactivation achieved for low‐ and high‐density populations of bacteriophage phi6 exposed in both SM buffer and artificial saliva using 50 mW cm^−2^ 405‐nm light is provided in Table 1. Samples were exposed to this higher irradiance for durations which resulted in the dose delivered being equivalent to that required to achieve complete/near‐complete (≥99.4%) bacteriophage inactivation when exposed to 0.5 mW cm^−2^ 405‐nm light (treatment dose [J cm^−2^] = irradiance [W cm^−2^] × time [seconds]). The mean germicidal efficiency (GE), defined as the log_10_ reduction in a bacteriophage population [log_10_(*N*/*N*_0_)] by inactivation per unit dose in J cm^−2^ (37) was calculated for bacteriophage inactivation under the various exposure conditions at both irradiance applications.

**Table 1:** Comparison of the log_10_ reduction and germicidal efficiency (GE) values associated with 405‐nm light inactivation of bacteriophage phi6 upon exposure to respective irradiances of 0.5 and 50 mW cm^−2^.

Exposure Conditions		Dose (J cm^−2^)	0.5 mW cm^−2^		50 mW cm^−2^	
			Log_10_ Reduction	GE	Log_10_ Reduction	GE
10^3^ PFU mL^−1^	SM Buffer	172.8	3.046 (±0.154)	0.018 (±0.001)	3.403* (±0.011)	0.020* (±0.000)
	Artificial Saliva	43.2	2.411* (±0.539)	0.056* (±0.012)	0.030 (±0.085)	0.002 (±0.002)
10^7^ PFU mL^−1^	SM Buffer	259.2	6.278* (±0.652)	0.024* (±0.003)	3.033 (±0.036)	0.011 (±0.000)
	Artificial Saliva	97.2	6.182* (±0.005)	0.064* (±0.005)	0.443 (±0.069)	0.004 (±0.001)

Each data point represents the mean value ± SD (*n* ≥ 4). Asterisks (*) represent values which are significantly higher than that of the other irradiance application (two sample *t*‐test; *P* ≤ 0.05, Minitab Statistical Software v19).

With the exception of low seeding density exposures in SM buffer, log_10_ reductions and GE values were significantly higher when exposed using 0.5 mW cm^−2^ as opposed to 50 mW cm^−2^ (*P* ≤ 0.05). At low seeding density in artificial saliva, exposure to 43.2 J cm^−2^ achieved a 2.41 log_10_ reduction with a GE value of 0.056 using 0.5 mW cm^−2^ light in comparison with just a 0.03 log_10_ reduction and 0.002 GE using 50 mW cm^−2^ light (*P* ≤ 0.05). Similarly, at high seeding densities, exposure to 97.2 and 259.2 J cm^−2^ in artificial saliva and SM buffer, respectively, resulted in greater inactivation and GE values when exposed using 0.5 mW cm^−2^ as opposed to 50 mW cm^−2^ (6.182–6.278 log_10_ reductions and GE values of 0.024–0.064 *versus* 0.443–3.033 log_10_ reductions and GE values of 0.004–0.011, respectively; *P* ≤ 0.05). This trend was however not apparent with the low‐density exposures in SM buffer, where similar (but significantly different) inactivation and GE values were observed between the two irradiances (3.403 log_10_ reduction and 0.020 GE for 50 mWcm^−2^ *versus* 3.046 log_10_ reduction and 0.018 GE for 0.5 mWcm^−2^; *P* ≤ 0.05).

## DISCUSSION

This study provides the first evidence demonstrating the efficacy of low irradiance 405‐nm ceiling‐mounted light systems for the inactivation of a SARS‐CoV‐2 surrogate, bacteriophage phi6, using parameters (irradiance and treatment distance) representative of practical system deployment. Importantly, results demonstrate the significant enhancement in phage susceptibility when exposed while suspended in artificial saliva: an important consideration due to the fact that the virus is expelled into the environment within respiratory secretions.

For environmental decontamination applications involving occupied areas, it is essential that low irradiance (<1 mW cm^−2^) 405‐nm light sources are employed such that the illumination produced is within the limits considered safe for continuous human exposure (38). For this study, exposures were conducted at approximately 1.5 m below the light source (using an irradiance of 0.5 mW cm^−2^), with these parameters selected as being representative of the illumination expected within high‐touch areas of a typical occupied public setting (36).

Based on the Hazard Group classification of SARS‐CoV‐2 (HG3) and the containment level of the laboratory utilized (BSL2), experimental testing on SARS‐CoV‐2 was not possible and so bacteriophage phi6 was instead employed as a coronavirus surrogate. Additionally, the phage densities used were representative of viral loads present in the saliva of individuals infected with SARS‐CoV‐2 at Day 0 (~10^7^ copies mL^−1^) and Day 24 (~10^3^ copies mL^−1^) post‐symptom onset, respectively (39). Phi6 is a dsRNA lytic bacteriophage of the Cystoviridae virus family which infects *Pseudomonas* bacteria (40, 41). It possesses similarities to that of coronaviruses, namely, it is of similar size, has spike proteins and is enveloped by a lipid membrane (40, 41, 42), and thus has been suggested and utilized as a surrogate for the study of SARS‐CoV‐2 in multiple publications (35, 42, 43, 44, 45, 46, 47, 48, 49, 50, 51).

The results of this study demonstrate bacteriophage phi6 can be successfully reduced when exposed in minimal SM buffer media; highlighting that 405‐nm light inactivation is attainable in the absence of exogenous photosensitizers, as previously demonstrated for SARS‐CoV‐2 or appropriate surrogates (30, 32, 34, 35). Due to their lack of porphyrins, viruses and bacteriophages demonstrate the lowest susceptibility of all microorganisms to 405‐nm light inactivation (26) and minimal inactivation has previously been indicated for non‐enveloped viruses unless exposed to very high doses or suspended in organically rich media (27, 28). However, Rathnasinghe *et al*. (30) recently demonstrated the significantly increased susceptibility of enveloped viruses in comparison with non‐enveloped viruses, hypothesizing that the lipid envelope may be able to absorb 405‐nm light wavelengths and contribute to the inactivation effect *via* either consequential ROS production instigating an oxidative effect, or simply destruction of the envelope. The results of this study can be considered to agree with this theory as, although significantly lower than that achieved when suspended in artificial saliva (*i.e*. in the presence of exogenous photosensitizers), statistically significant 0.06 and 0.83 log_10_ reductions of low‐ and high‐density populations, respectively, were observed when exposed in SM buffer, after 24 h exposure (*P* ≤ 0.05); suggesting the inactivation effect is likely somewhat accountable to light interactions with the phage envelope. In addition, the doses required for a 1 log_10_ reduction of high‐density populations were found to be within the same orders of magnitude of that previously established for bovine coronavirus as a surrogate for SARS‐CoV‐2 exposed in phosphate‐buffered saline, which is also absent of photosensitive material (57.5 J cm^−2^ in comparison with 43.2 J cm^−2^ found in this study) (34). Further investigation into the interactions of viral envelopes with 405‐nm light, although beyond the scope of this study, will be essential in advancing understanding of the viricidal efficacy of 405‐nm light.

This study additionally evaluated the potential enhancement in phage susceptibility when exposed in artificial saliva; selected due to saliva representing a significant vector media in the transmission of SARS‐CoV‐2 (52). The mucins of saliva (or porcine stomach mucins as substituted in this study) contain light‐sensitive chromophores that are likely predisposed to 405‐nm light photosensitization, and the potential for enhancing viral inactivation to 405‐nm light has been previously demonstrated (28). The hypothesis is that the photosensitive components within nutrient‐rich media, such as saliva, can act as exogenous photosensitizers, absorbing the 405‐nm photons and initiating Type‐I and Type‐II photodynamic reactions resulting in the local release of ROS which can impart oxidative damage to viral and phage structures in close proximity (27, 28). The results of this study are consistent with this theory, with 405‐nm light inactivation significantly enhanced when exposed in artificial saliva compared to when in SM buffer: 83.3–87.5 and 50% less dose was required for a 1 log_10_ reduction of phi6 at low‐ and high‐seeding densities, respectively. These results are comparable with that of previous findings (27, 28), which demonstrated 88–89% less dose was required for a 1 log_10_ reduction in both feline calicivirus and phage φC31, at similar population densities of 10^5^ and 10^3^ PFU mL^−1^, respectively, when exposed to 405‐nm light in organically rich media in comparison with minimal media. The dose requirements were significantly higher for feline calicivirus and φC31 in comparison with phi6 in this study (27, 28), however, it should be noted that both feline calicivirus and φC31 are non‐enveloped and the increased susceptibility of enveloped *vs* non‐enveloped viruses and bacteriophages to visible light inactivation is previously described (53). It is also of interest to note that the authors utilized significantly higher irradiances for viral and phage exposure than those employed here (155.8 and 56.7 mW cm^−2^, respectively (27, 28)), suggesting this enhancement effect is apparent regardless of the light delivery method.

Whereas it is to be expected and shown that use of higher irradiance 405‐nm light levels will increase the rate of microbial inactivation (54), the data in this paper establish that lower levels of 405‐nm light are actually more germicidally efficient. The log_10_ reductions and GE values shown in Table 1 indicate that lower irradiance 405‐nm light is more efficient on a per unit dose basis for phage inactivation in comparison to that of equivalent higher irradiance exposures. At high‐seeding densities, 0.5 mW cm^−2^ exposures achieved 2.07–5.79 greater log_10_ reductions and 2.18–16 times greater GE values than that of 50 mW cm^−2^ exposures. Similarly, in artificial saliva at low‐seeding densities, 0.5 mW cm^−2^ exposures achieved a 2.35 greater log_10_ reduction and a 28 times greater GE value than that of 50 mW cm^−2^ exposures. This trend was not demonstrated for low‐density exposures in SM buffer, with similar inactivation and GE values observed: this is most likely due to the fact that the time to apply this required dose using 50 mW cm^−2^ light (57.6 min) was, in this case, still sufficient to achieve complete inactivation of low‐density populations and it is likely that sub‐lethal doses would elucidate greater variation. In addition, non‐exposed equivalent controls, from which log_10_ reductions and GE values were calculated, were lower for the 0.5 mW cm^−2^ exposures due to the prolonged nature of treatment, as previously discussed (27).

The enhancement in susceptibility observed with lower irradiance treatments is consistent with data in previous studies. Vatter *et al*. (35) exposed a 10^7^ PFU mL^−1^ phi6 population in SM buffer to 405‐nm light at a higher irradiance of 78.6 mW cm^−2^ and the dose required to achieve a 3 log_10_ reduction was significantly higher than that required in this study: approximately 1300 J cm^−2^ in comparison to approximately 129.6 J cm^−2^. This is further corroborated by the recent findings of Rathnasinghe *et al*. (30), who demonstrated that 405‐nm light at a lower irradiance of 0.035 mW cm^−2^ could achieve a 1.03 log_10_ reduction in 10^5^ PFU mL^−1^ SARS‐CoV‐2 following 24 h exposure (3.024 J cm^−2^); a lower dose than that required at 0.5 mW cm^−2^ for similar 1 log_10_ reductions of 10^3^ PFU mL^−1^ populations (>129.6 J cm^−2^) and 10^7^ PFU mL^−1^ populations (43.2 J cm^−2^) in this study. The results of the present study, both independently and when compared to the results of relevant earlier studies, provides fundamental evidence of the enhanced susceptibility (per unit dose level) of bacteriophage phi6 to 405‐nm light when exposed at lower irradiances, further strengthening the proposal of visible violet‐blue light systems for environmental decontamination of SARS‐CoV‐2. Further investigation into the associated photochemical mechanisms involved in 405‐nm light inactivation of bacteriophage phi6 is required to further elucidate these findings and augment clinical translatability of this technology.

Overall, this study has successfully demonstrated the ability of low irradiance antimicrobial 405‐nm light systems to inactivate bacteriophage phi6 populations at both low‐ and high‐seeding densities in both minimal SM buffer and biologically relevant artificial saliva, with susceptibility to inactivation significantly enhanced when suspended in artificial saliva, which is known to possess photosensitizers. The exposure conditions used were chosen to replicate those employed for the decontamination of whole‐room environments and these findings are therefore of significant interest as they enhance understanding of the antimicrobial capabilities of low irradiance 405‐nm light systems and, in conjunction with its established safety benefits, further the potential of this technology to be used as a novel approach to achieve widespread decontamination within occupied settings and help tackle environmental transmission of COVID‐19. An evaluation of the efficacy of 405‐nm light for the inactivation of SARS‐CoV‐2 without the use of a surrogate, across a greater range of irradiances likely to be produced by these systems in a typical room setting, will be essential to further implementation of low‐irradiance 405‐nm light systems for environmental decontamination purposes.

## Data Availability

Data supporting this publication are stored by the University of Strathclyde. Details of the data and how it can be accessed are available from the University of Strathclyde KnowledgeBase at https://doi.org/10.15129/2d8a231a‐84de‐40bf‐9345‐b907c67affc6.
